# Global Voices, Shared Futures: Early-Career Scientists on the Power of Collaboration

**DOI:** 10.1021/acscentsci.5c01854

**Published:** 2025-11-26

**Authors:** Krystyna Maslowska-Jarzyna, Diego Gomez-Maldonado, Bryan D. James, Ty Christoff-Tempesta, Katharina Ehrmann, Eleonora Comeo, Susmita Sarkar, E. Celeste Welch, Jianyu Zhang

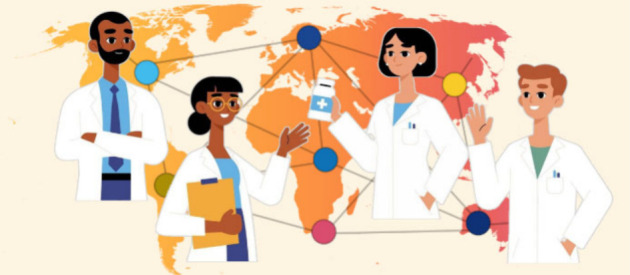

Solving the world’s greatest challenges, from climate change to public health, requires more than brilliant ideas. It requires people who can work together across borders, disciplines, and institutions.^1,2^ In recent years, international academic exchange has faced growing political and administrative barriers, often argued for national security reasons.^3,4^

In 2023, we—a diverse international authorship team of scientists, representing various chemistry subdisciplines—came together through the American Chemical Society’s CAS Future Leaders program,^5^ which aims at connecting and empowering early-career scientists to take on leadership roles and impact the future of the chemical sciences.

In this editorial, we draw on our collective experiences and peer reflections guided by three questions:

(1) Why are global scientific connections necessary?
(2) How can society benefit from these connections?
(3) How can we influence the future of collaborations to deliver real-world solutions?

We hope this text will initiate dialogue and encourage investment in international collaboration. Full responses from the authors and 2023 cohort members can be found in the Supporting Information.

## 1 The Value of Global Connections

Collaborations play a pivotal role in shaping our research and identity as scientists. They catalyze innovation and drive scientific progress. Building collaborations, especially as early-career researchers, requires initiative and persistence in the face of challenges, but also rewards us with a sense of connection, a shared purpose, a support network, and space to grow personally and professionally.

### 1.1 Collaborations Make Better Scientists

By bringing together diverse perspectives, constructive feedback, and new methodologies, collaboration enables researchers to address complex problems that are difficult to solve in isolation. The results of collaborative work are more than the sum of the individual researchers, as they create a product that leverages unique strengths and expertise of each contributor. One of the most widely recognized values of such synergetic collaboration is the ability to conduct interdisciplinary research, bridging the gap between disciplines and accelerating scientific discovery. For early-career scientists, in particular, collaboration offers not only intellectual enrichment but also solidarity, recognition, mentorship, and a sense of belonging. Ultimately, collaboration not only yields better science but also fosters better scientists.

Scientific collaborations foster both personal and professional growth by strengthening essential interpersonal skills, such as communication, teamwork, and project management—qualities essential for careers in academia, industry, government, or nonprofit sectors.

### 1.2 Building Collaborations

There are many creative ways to build collaborations: through conferences, cold emails, colleagues, or structured programs. While the process might seem intimidating, many people are eager to share their knowledge and start a collaboration. Establishing collaborations comes, however, with its own set of challenges, e.g., the need to coordinate across time zones, secure independent funding, or navigate the early stages of unfunded projects. Still, certain factors consistently emerged as key to making collaborations successful: the foundation of successful collaborations lies in shared interests, complementary skills, a willingness to invest time in a common goal, trust, and, like in any good relationship, open communication!

### 1.3 The Rewards of Collaboration

Collaborations offer far more than just scientific output. The most immediate results are joint publications, conference presentations, access to new methodologies, or insights into other research fields. But collaborations also bridge personal and professional growth through the formation of friendships and mentorship relationships, which can ultimately become lasting networks.

Collaborations also have an emotional side, which highlights the importance of patience, humility, cross-cultural understanding, and being open to not having all the answers. Collaboration allows us the opportunity to lean on the strengths of others and avoid reinventing the wheel. A longer term reward comes from broadening our perspective and changing our approach to science. Naturally, not every collaboration leads to immediate success, but even challenges can be formative. Ultimately, the most profound lesson from collaboration is not just what you learn from others, but how you grow and incorporate this knowledge with your existing expertise.

## 2 Connecting Science and Society

Working across disciplines and borders teaches us to share our research with clarity, purpose, and empathy. In this section, we reflect on how collaboration informs the way we make science accessible and why it is important to connect science with society.

### 2.1 Clear Communication Is Key

Interdisciplinary collaboration has made us aware of the jargon we use daily and teaches us how easily misunderstandings can arise when we assume shared knowledge. Effective collaboration requires, therefore, the ability to adapt language, appreciate the broader context, and frame science in general terms that have intelligible meaning beyond the confines of a narrow specialization. Storytelling, contextualization, and analogies are effective strategies for improving science communication.

### 2.2 Making Science Accessible beyond the Lab

The responsibility for effective communication extends well beyond professional circles. Scientists are increasingly being called upon to share their work with wider audiences, whether by teaching students, engaging policymakers, explaining research to children, families, and community groups, or creating online content for social media. These forms of communication combine factual knowledge with narrative and can be an important tool for public education and implementing structural changes. However, communicating science across disciplines and levels of background knowledge requires clarity, empathy, and the ability to adapt one’s language to the target audience. Visuals, metaphors, analogies, and framing serve as bridges that link abstract concepts to everyday life, sparking curiosity and encouraging participation.

Ultimately, effective outreach is not about oversimplifying science, but about connecting with the knowledge and values people already hold.

### 2.3 The Societal Value of Accessibility Is Profound

Clear and open communication is the foundation of the bond between science and society. It builds public trust, empowers informed decision-making, and helps combat misinformation in critical areas like health, technology, and environmental responsibility. By making research transparent and approachable, scientists demystify it, showing that science is not confined to laboratories or institutions, but can be accessible, relevant to everyday life, and directly connected to social well-being.

Importantly, scientific accessibility is not only a service to society but also helps to strengthen scientific research itself. Research that is communicated understandably is more likely to spark innovation, foster interdisciplinary collaborations, and inspire the next generation of scientists. In this sense, communication becomes part of the research process.

In public funding, accessibility is both a professional duty and a moral responsibility: society invests in science and should benefit from its knowledge. From a broader perspective, accessible science contributes to technological progress and economic development, as well as civic literacy, cultural growth, and the sustainable future of humanity.

## 3 The Future of Collaboration: A Call for Action

To look ahead, we asked the Future Leaders cohort: “*What do early-career scientists need to build better collaborations?*” The responses reveal a coherent sense of optimism that early-career researchers are already laying the foundations of a connected, inclusive, and impactful scientific community. But we cannot do it alone. To fully unlock the potential of these grassroots efforts, we need structural support, space for long-term dialogue, and a commitment to collaboration that goes beyond institutions and borders.

### 3.1 Strategies for Creating Collaborations

Early-career scientists build collaborations through intentional networking, mutual support, and sustained informal exchange. Creating meaningful partnerships often goes beyond one-time encounters. Efforts to continue initial discussions and plans can take the form of postconference follow-ups. Likewise, long-term relationships with peers from previous internships, research experiences, courses, or leadership programs can be cultivated by creating opportunities continued virtual or in-person dialogue. For example, quarterly check-ins can do wonders for maintaining a connection to peers over time.

This editorial is itself an example of a grassroots initiative, demonstrating that collaboration begins with connection, not titles. Bringing together early-career scientists across continents, without institutional support, hierarchy, or formal funding, represents a unique way to collaborate as equals driven by a shared purpose rather than obligation. Digital spaces and professional communities also foster collaboration beyond disciplinary boundaries. We can leverage these networks to host and engage in initiatives such as webinar series on topics such as equity, diversity, and inclusion. It must be emphasized that collaboration does not always begin with a research project. It might start with giving feedback, exchanging experiences, or simply asking questions. The key to a meaningful collaboration is to find a mutually beneficial situation for all involved, maintain communication, and exercise patience.

### 3.2 Personal and Societal Benefits of Collaborations

Collaborations between scientists help to overcome knowledge gaps, streamline research, and make work more impactful. The benefits extend well beyond individual laboratories. For example, diverse collaborations can help to promote the chemical sciences as a viable and rewarding career for underrepresented groups, thus building a more inclusive scientific community. This inclusivity fuels innovation and strengthens the pipeline of future scientific leaders. For society, these collaborations lead to faster scientific progress to solve pressing real-world problems. Coming together, we can more efficiently generate new solutions to the biggest challenges in society, such as those related to climate change, sustainability, and human health.

### 3.3 Dreaming Big: Building Your Network

Early-career scientists envision bold, creative ways to expand their collaborative networks, both within and beyond academia. Conferences remain key, but there is a strong desire for their traditional format to be reorganized into more inclusive and interactive spaces that promote interactions between attendees. Smaller, student-focused events such as department-level symposia across specialties can also be highly useful in helping students develop skills and confidence to network effectively. Digital platforms also offer new possibilities, e.g., interactive databases to match collaborators by expertise or social media, as a tool for forming global connections. Still, the most transformative collaborations often begin with genuine, sustained personal contact—while you can read someone’s papers for years, sharing a lab bench changes everything, and this is where mobility programs come in.

To turn these approaches into practice, we need targeted support: seed funding, institutional flexibility, and infrastructure for long-term dialogue. Interdisciplinary workshops, online forums, and collaborative projects could spark cocreation among scientists and nonscientists alike. In addition to resources, a collaboration-supportive mindset is important.

The modern research environment often focuses on the independence of younger researchers, especially when applying for grants or fellowships.

Applicants are frequently expected to provide evidence of leading publications without their supervisors or establishing “independent” lines of research distinct from their previous mentors. While we acknowledge that collaborations can connect loosely related fields, we believe that the collaborative work of experts in the same field could not only accelerate progress but also develop it beyond achievements accessible to separate research efforts. We further suggest that systems such as the diligent use of CRediT statements^6^ in research articles will allow for transparent accreditation of contributions. These ideas point to a shared aspiration: a more connected, collaborative, and human-centered scientific future.

While these expectations aim to support career progression, they can inadvertently discourage meaningful collaboration, especially with peers in similar fields!

Looking ahead, while mobility and dialogue remain essential to building trust in science, new technologies, especially artificial intelligence (AI), are rapidly expanding the possibilities for global collaboration by breaking down traditional barriers.^7^ Tools like DeepL and Google Translate support real-time multilingual communication, while platforms such as Semantic Scholar, Elicit, and ChatGPT accelerate literature reviews and idea generation across disciplines. As AI becomes more embedded in research workflows, it accelerates discoveries, and calls for globally coordinated efforts around equitable access, data sharing, and ethical governance.

As we reflect on the impact of our experiences and the collaborations we have built, we are reminded that science transcends borders, politics, and language. Strengthening scientific exchanges is conducive to the growth of human knowledge and technological progress. Still, several countries and institutions have aimed to prevent international academic exchanges and study abroad cooperation in the name of national security. The CAS Future Leaders is a project that breaks this kind of limited academic exchange. In a world where global cooperation is increasingly challenged, this vision of open exchange is more vital than ever.

In summary, we believe that global and interdisciplinary collaborations are important for conducting scientific research with maximum societal impact. These relationships can help us to learn from and leverage the strengths of others. Scientific collaborations go beyond simply finishing independent parts of projects; they enable us to develop new skills, broaden our horizons, and acquire unique research expertise. They ultimately confer a greater sense of personal utility and belonging. Furthermore, effective scientific communication and outreach are essential for advocating our science and conducting research with maximum benefit, translating fundamental discoveries and conferring them with greater real-world relevance. Now more than ever, it is essential to recognize the need for breaking out of narrow academic silos and promoting enhanced collaboration and communication within the broader scientific landscape.

## Supplementary Material



## Notes

Author biographies, full responses to questions from authors and fellow cohort members (PDF)
