# Supplementary material for: Global Voices, Shared Futures: Early-Career Scientists on the Power of Collaboration
Source: ACS Cent Sci. 2025 Nov 26;11(11):2018–21. doi: 10.1021/acscentsci.5c01854 (PMC12670316; doi:10.1021/acscentsci.5c01854)
Supplement: Supplementary file 1 [file oc5c01854_si_001.pdf]

Supporting Information

Global Voices, Shared Futures: Early-Career Scientists on the Power of Collaboration

Krystyna Maslowska-Jarzyna,[a,*] Diego Gomez-Maldonado,[b] Bryan D. James,[c] Ty Christoff-Tempesta,[d] Katharina Ehrmann,[e] Eleonora Comeo,[f] Susmita Sarkar,[g] E. Celeste Welch[h] Jianyu Zhang[i]

AUTHOR ADDRESS: (a) University of Warsaw, Faculty of Chemistry, Biological and Chemical Research Centre, 02-089 Warsaw, Poland; (b) Texas Tech University, Fiber and Biopolymer Research Institute, Department of Plant and Soil Sciences, Lubbock, TX 79403, USA; (c) Department of Chemical Engineering, Northeastern University, Boston, MA 02115, USA; (d) University of California, Irvine, Department of Materials Science and Engineering, Department of Chemical and Biomolecular Engineering, CA 92697, USA; (e) Technische Universität Wien, Institute for Applied Synthetic Chemistry, 1060 Wien, Austria; (f) University of Nottingham, Division of Biomolecular Sciences and Medicinal Chemistry, Biodiscovery Institute, School of Pharmacy, Nottingham NG7 2RD, United Kingdom; (g) North Carolina State University, Raleigh, Department of Mechanical and Aerospace Engineering, NC 27695, USA; (h) Stanford University, Department of Chemical Engineering, Stanford, CA 94305, USA; (i) State Key Laboratory of Biobased Transportation Fuel Technology, Department of Polymer Science and Engineering, Zhejiang University, Hangzhou 310058, P.R. China.

*Corresponding author: kmaslowska@chem.uw.edu.pl

TABLE OF CONTENTS

AUTHOR BIOGRAPHIES S3
Q&A S7
1. The value of global connections S7
2. Connecting science and society S12
3. The future of collaboration – a call for action S18
APPENDIX S24
Programs that support researcher mobility and international collaboration S24

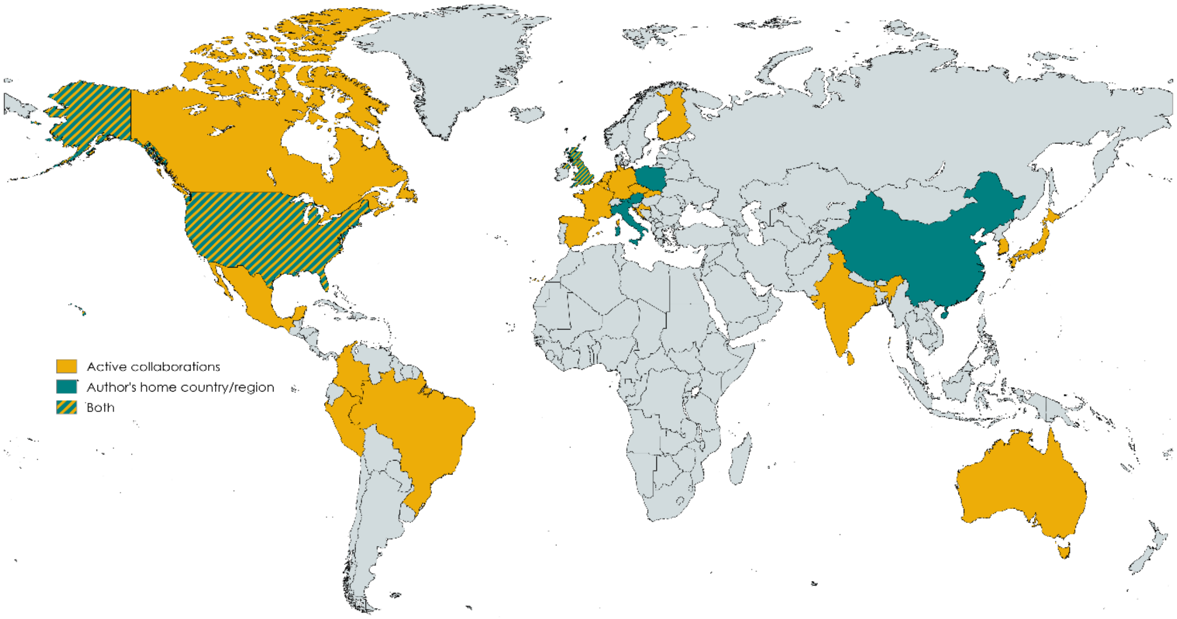

Figure S1. Geographic distribution of authors' home countries and their documented international research collaborations. Teal indicates authors' home countries; yellow marks countries involved in collaborations; striped areas represent overlap. The map was created with mapchat.net.

AUTHOR BIOGRAPHIES

Krystyna Maslowska-Jarzyna (University of Warsaw)

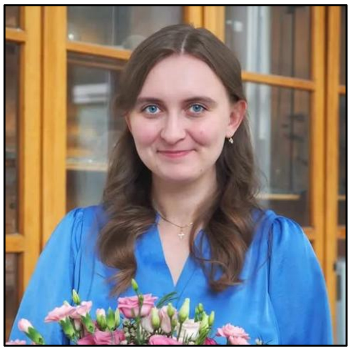

Dr. Krystyna Maslowska-Jarzyna is a senior assistant at the Faculty of Chemistry, University of Warsaw (Poland). She completed her BSc, MSc, and PhD at the Biological and Chemical Research Centre (UW) in the group of Prof. Michał Chmielewski. Her research interests focus on the supramolecular chemistry of anions, particularly the transport of biologically relevant anions across lipid bilayers using synthetic molecular carriers. During her studies, she completed research internships at Tulane University (USA), the University of Cagliari (Italy), the Université libre de Bruxelles (Belgium), and the University of Porto (Portugal). After earning her PhD in 2024, she joined the group of Prof. Phil Gale at the University of Technology Sydney (Australia) as a postdoctoral fellow, where she worked on organelle-targeting anion transporters. Her research has been recognized with multiple awards and fellowships, including the CAS Future Leaders Award, the Bekker Fellowship from the Polish National Agency for Academic Exchange (NAWA), the START Fellowship from the Foundation for Polish Science, the Świętosławski Award for scientific achievements, the Kościuszko Foundation Grant, and a national scholarship from the Minister of Science and Higher Education. Outside the lab, Krystyna is passionate about travel and has visited over 40 countries. Her interests also include creative projects, science communication, mentoring young researchers, and promoting inclusive scientific collaboration.

Diego Gomez-Maldonado (Texas Tech University)

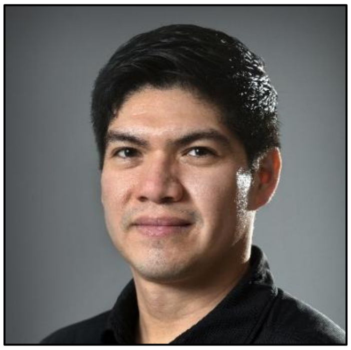

Dr. Diego Gomez-Maldonado is the PI at the Bio Nano Advanced Health Materials at the Fiber and Biopolymer Research Institute at Texas Tech University. His research focus is on the development of advanced sustainable and renewable materials for health applications. Prior to this, Diego did postdoctoral work at Northeastern University researching the microenvironment of neural cells for regenerative medicine and tissue engineering. He holds a PhD in Forest Biomaterials from Auburn University, a M.Sc. in Material Design and Engineering, and a B.S. in Biological Engineering both from Universidad Autonoma Metropolitana in Mexico. He has expertise in nanomaterials and biopolymer surface chemistry, characterization, and 3-D assembly. Equally important is his interest in education and mentoring as tools to harvest a sense of belonging and to build a sustainable future. Outside of research, Diego enjoys reading fiction novels and X-Men comics, walking out in town, and going to the movies.

Bryan D. James (Northeastern University)

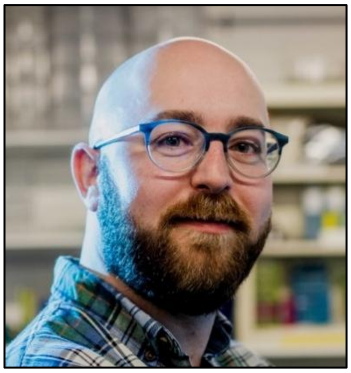

Dr. Bryan D. James is an assistant professor in the Department of Chemical Engineering at Northeastern University. He completed his postdoctoral studies at the Woods Hole Oceanographic Institution and his graduate studies at the University of Florida. His research group designs functional, sustainable, and benign materials and chemicals, with particular emphasis on plastics, and develops tools to support decision-making in engineering design. Outside of the lab, Bryan can be found hiking, grilling, or catching the latest movie in theatres.

Ty Christoff-Tempesta (University of California)

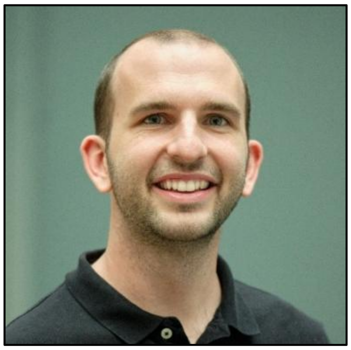

Dr. Ty Christoff-Tempesta is an Assistant Professor and Samueli Faculty Development Chair in the Department of Materials Science and Engineering at the University of California, Irvine. He received his BS in Materials Science and Engineering from the University of Florida and his PhD from the Massachusetts Institute of Technology in Polymers and Soft Matter. His lab investigates how molecular designs emerge unusual properties in organic materials to address outstanding challenges in sustainability and health, including plastics waste, water treatment, and climate change. Outside of his academic life, Ty enjoys cooking, playing volleyball, and hiking.

Katharina Ehrmann (Technische Universität Wien)

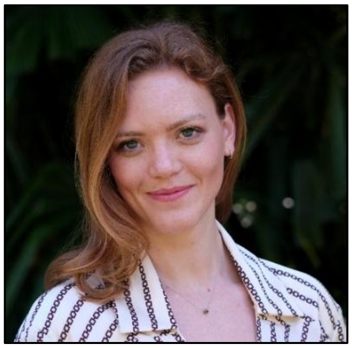

Dr. Katharina Ehrmann is an Elise Richter Fellow at the Institute of Applied Synthetic Chemistry (TU Wien), pursuing Habilitation and leading the Additive Manufacturing Team at TU Wien. She is working on broadening the processing window of and rethinking the chemistry behind light-based additive manufacturing (3D printing) of polymers to obtain high-performance parts with functionality. In particular, she is interested in multi-wavelength and multi-temperature 3D printing to obtain multi-material objects. She studied Chemistry at the University of Innsbruck (Austria) and the University of Edinburgh (UK). During her PhD in the group of Prof. Robert Liska at TU Wien (Austria) Katharina developed self-reinforcing thermoplastic polyurethanes for tissue engineering applications. She then became a postdoctoral research fellow at the Queensland University of Technology (QUT, Australia) in 2021, where she worked on wavelength-resolved photopolymer-networks in the group of Prof. Christopher Barner-Kowollik and has remained a visiting research fellow at QUT since her return to TU Wien in 2023.

She is also a member of the International Younger Chemists Network (IUPAC) and a Member-At-Large in the Polymer Materials Science and Engineering (PMSE) devision of the American Chemical society (ACS), and has received several prestigious prizes and fellowships, such as the Dr. Ernst Fehrer Prize, Chemistry Europe Travel Exchange Grant, CAS Future Leaders fellowship, and the FFG Female Innovator fellowship. Beyond her academic research, Katharina is passionate about outreach and organizes the Kids' Participation Labs and courses in the Kids' University programme for hands-on science education.

Eleonora Comeo (R&D company)

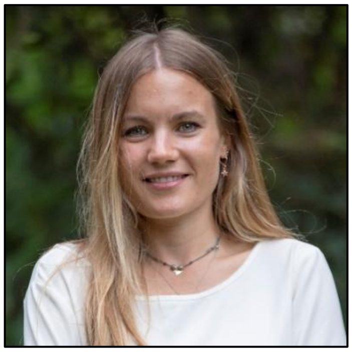

Dr. Eleonora Comeo received her M.Sc. degree in chemistry and pharmaceutical technologies at the Alma Mater Studiorum University of Bologna in 2016. She then pursued her Ph.D. degree in molecular pharmacology and drug discovery joint between the University of Nottingham and Monash University. Her doctoral studies focused on the design, development and application of fluorescently-labelled molecules to study the biology of membrane proteins in living cells. Following her PhD, Eleonora continued as research fellow in medicinal chemistry working in Prof. Michael Stocks' lab, where her work was focused on designing and synthesizing new drug-like molecules and chemical probes targeting the P2Y2 receptor. She then moved to Industry, working as a scientific project manager in R&D for a healthcare company that is pioneering the design, development, and production of 100% natural and biodegradable products for health and well-being. Beyond her work, Eleonora enjoys travelling, doing sport (running and tennis), collecting vinyl records and reading sci-fi and drama books.

Susmita Sarkar (North Carolina State University)

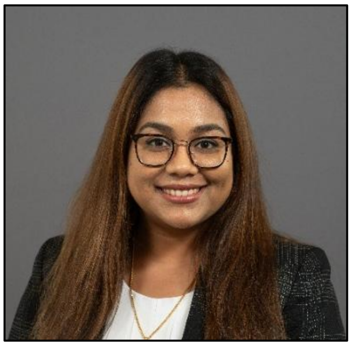

Dr. Susmita Sarkar is an Assistant Professor in the Department of Mechanical and Aerospace Engineering at North Carolina State University. She earned her Ph.D. from Purdue University and completed a postdoctoral fellowship as a Schmidt Science Fellow at Stanford University. Her research focuses on next-generation batteries, with particular emphasis on lightweight, high-performance energy storage for eVTOLs and soft, stretchable batteries for biomedical applications. Outside the lab, she mentors student-led innovation teams, including SolarPack, and enjoys promoting science outreach and interdisciplinary collaboration.

Cel Welch (Stanford University)

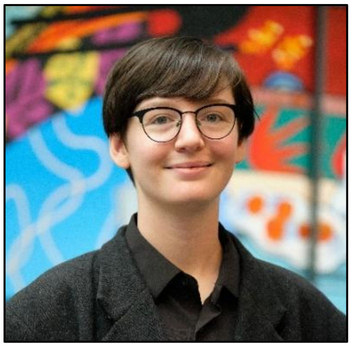

Dr. E. Celeste (Cel) Welch is a postdoctoral researcher in Zhenan Bao's group in the Stanford University Department of Chemical Engineering. Cel received a PhD and ScM from Brown University in Biomedical Engineering and a BSc from McGill University. They are currently developing flexible electronic devices for integration, sensing, and control of tissues. Broadly, Cel is interested in developing mico/nanodevices and using bioelectronics to fundamentally understand and leverage cellular bioelectricity. Outside of the lab, Cel enjoys running and reading.

Jianyu Zhang (Zhejiang University)

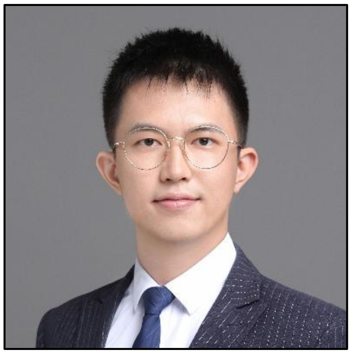

Dr. Jianyu Zhang is a tenure-track ZJU100 professor at Zhejiang University, China. He obtained his BSc in Chemistry from Sun Yat-sen University in 2019 and PhD degree from The Hong Kong University of Science and Technology in 2022. Later, he was a postdoctoral fellow at HKUST with Prof. Ben Zhong Tang and a research fellow under the Marie Skłodowska-Curie Actions with Prof. Ben L. Feringa at the University of Groningen in the Netherlands. His research interests include organic luminescent materials, clusteroluminescence, through-space conjugation, dynamic chemistry, and computational chemistry for excited-state evolutions. He has published 108 articles, including 37 first authored and 4 corresponding authored publications in *Nat. Photon.*, *J. Am. Chem. Soc.*, *Nat. Commun.*, *Angew. Chem. Int. Ed.*, etc. Dr. Zhang was listed in the World's Top 2% Scientists 2024. In addition, he has received many renowned honors, including ACS PMES Future Faculty Award (2025), ACS PHYS Young Investigator Award (2024), CAS Future Leaders (2023), Marie Skłodowska-Curie Actions Fellowships (2023), Hong Kong RGC Postdoctoral Fellowship (2023), and HKUST Academic Excellence Award (2022).

Q&A

1. The value of global connections

1.1. What value do you find in scientific collaborations?

Hirogi: Scientific collaborations are invaluable because they bring together diverse perspectives, expertise, and resources to tackle complex problems that no single researcher or institution could solve alone. In particular, insights from non-specialist fields often lead to fresh, unconventional ideas that specialists might overlook due to disciplinary blind spots. These unexpected perspectives can spark groundbreaking innovations.

Monika: Collaborations allow for approaching problems from different angles, which is important in so many ways - from the set-up of a scientific question and experiment, to the way that data is interpreted and solutions are innovated.

Croix: In my view, scientific collaborations are one of the key drivers of scientific progress. The value of collaborations comes in the different perspectives individual scientists bring to a collaboration—differing perspectives and experiences influence how observations are interpreted, how scientific questions are approached.

Azalea: Scientific collaborations offer numerous benefits. They accelerate research by enabling knowledge sharing across laboratories and providing access to specialized equipment or instruments that may not be available in-house. Collaborations also foster creativity—working with a diverse group of researchers often leads to innovative approaches to problem-solving. From a graduate student's perspective, collaborations are also excellent training grounds for developing interpersonal skills such as communication, teamwork, and project management. These skills are essential for careers in academia, industry, government, or non-profit sectors.

Krystyna: Collaboration is no longer an optional "add-on" to research, but it is the foundation of my development as a scientist. Working across borders and disciplines enriches science and changes the way we do it. But collaboration is not just about knowledge – it is also about solidarity. As early-career researchers navigating a demanding and often unstable system, we need a community of trust and support. In my experience, collaboration builds resilience, and reminds us that we don't have to figure everything out alone.

Katharina: Novelty often emerges where two research fields meet. This requires us to look beyond our own expertise and collaborate with other fields. Truly collaborative work gives rise to more than the sum of the individual researchers because they inspire each other.

Cel: Scientific collaborations can be helpful to leverage multiple diverse expertises. This can produce work that is high quality and multidisciplinary in nature.

Diego: I think it generates both a support systems and stronger science by complementing expertise.

Jianyu: Complementarity of skills and technology, exchange of academic thoughts, collision of ideas.

Bryan: Science cannot be done in a vacuum! Collaborations are imperative.

Roxana: Scientific collaborations enable brainstorming of complex ideas that facilitate research progress. They can also promote interdisciplinary research that can drive innovative and sustainable solutions.

Eleonora: Collaboration in Science is the catalyst of innovation, it fosters a greater understading and knowledge of scientific problems which would not be possible by scientists, individuals in isolation. This is true for every facet and discipline in science. As drug hunter by training and at heart, I have found that team science approach is crucial for advancing drug discovery and development. As medicinal chemistry is a discipline located at the interface of chemistry and biology, collaboration bridges the gap between these disciplines and all those linked with them, thereby accelerating scientific discovery with real impactful solutions for changing the lives of patients.

Melissa: Scientific collaboration is critical to my research area wherein I combine organic synthesis and computation to enable the discovery and development of new chemical reactivity.

Sivani: The exchange of ideas, bringing in different perspectives, especially in an interdisciplinary context.

Susmita: I see scientific collaboration as a powerful antidote to the isolation that often comes with academic research, especially during graduate school, which can be a lonely and siloed process. One of the most formative experiences for me was during my PhD, when our lab collaborated with a national lab. Although the project wasn't directly mine, I found myself drawn to the weekly discussions that included external mentors. These meetings brought fresh perspectives, challenged assumptions, and created a much more dynamic and engaging scientific environment than working in isolation. That experience shaped how I now approach research as a PI: I actively seek collaborations that foster not just productivity, but intellectual community.

1.2. How have you built collaborations, and what made them successful/what challenges have you encountered along the way?

Hirogi: I have been involved in two international collaborative research projects, both of which began after in-person meetings and discussions. I believe that one of the main challenges in international collaborations is the difficulty in coordinating schedules across different time zones, which often makes it hard to have frequent discussions. Another significant challenge was the process of sending samples across borders, which involved complex procedures and strict reviews.

Monika: When I was in grad school, I worked with a community group, who wanted to understand more about an issue. We formed a collaborative working group and I applied for and was awarded a grant to pursue the project. The project was successful because it was driven by a need from the community and by community voices informing the direction of the project. Myself and others from the university provided the resources, expertise, and feedback from the experiments, which further fueled the conversation with the community and made the project successful.

Croix: When I was in academia, I mainly built collaborations by attending conferences and talking to people. I've observed that many collaborations are borne out of friendships, which seems like a natural progression. Affinities between individuals will lead to discussing what they study and, from that friendship, ideas will sprout and bloom. Now, in industry, I naturally collaborate less outside my institution just by the nature of my industry. However, companies function much like large-scale collaborations. Building healthy, professional relationships with my colleagues seems key to maintaining the company's success.

Azalea: During my PhD, I collaborated on three different projects with researchers from the Institute of Science Tokyo, Cornell University, and Stanford University. These collaborations were successful due to

open communication regarding project roles and timelines. At the beginning of the collaboration, I always established clearly defined roles and set rough check-in points for each project stage. As the project evolves, we adjusted timelines and responsibilities accordingly. Maintaining this open and flexible communication is key to managing progress and ensuring success.

Krystyna: Most of my international collaborations began through direct outreach by emailing researchers. During my PhD, I arranged three international research stays (Université libre de Bruxelles, University of Cagliari, and Tulane University), and for my first postdoc, I went to the University of Technology Sydney. What makes a collaboration successful, in my experience, is complementary skills, mutual curiosity and trust. The biggest challenge, especially across countries, is often timing and funding. Everyone is busy, and aligning goals and expectations takes effort. But when it works, the results are worth it, both scientifically and personally.

Katharina: I have connected with other researchers over extended research stays and at conferences. Collaborations strain resources particularly in the beginning, where no funding can be allocated directly to the collaboration. This drains the intensity at which the collaboration can operate and thereby succeed.

Cel: I have built some cross-institutional collaborations at conferences through networking with other researchers in my research area. Smaller conferences (e.g. Gordon-style) are a bit better for this, in my experience. Some challenges I have encountered are keeping the motivation going for the collaboration - communication will always be more challenging, particularly with things like time differences.

Diego: I think a great way to build collaborations is through assisting and getting immersed in scientific conferences and the social events that come from there. The biggest challenge for me is to transform chat to action.

Jianyu: For international cooperation, I once worked with a professor at the University of Copenhagen in Denmark, where the complementary skills of both parties (the other party provided new materials, and I developed new properties and applications) and smooth communication of progress were the keys to success. For domestic cooperation, I mainly engaged in theoretical computational chemistry and cooperated with researchers in various fields such as materials and biology. The most important thing was that neither party had the proficient skills of the other party, so that we could achieve complementarity, complete a high-quality project and publish a high-level paper.

Bryan: I've built collaborations through meeting people at workshops, cold emails, and through colleagues. They have been successful because of our shared interests and time spent building a personal connection. The conversations cannot just be science! The challenges I have experienced are when deadlines come up and expectations are not met. Like any good relationship, clear communication is key.

Roxana: I’ve been fortunate to build collaborations by networking at conferences. My PI has also leveraged their network to reach out to collaborators that I was interested in working with. I have also sent ‘cold’ emails to potential collaborators to ask if they would be willing to participate in a research project.

Eleonora: I have started with researchers working in my research field, known by reading papers simply trying reaching out with them. It's great that on the "other" side of the computer I have always found collaborative people, eager to share their knowledge and start a collaboration. During my PhD I have also

had the opportunity to spend one year abroad in Australia which clearly help in forging new collaborations and friendships along the way, also with scientists working on different chemical & life sciences subjects. I think a possible key to successful collaboration is the willingness to really understand and appreciate someone'else perspective and approach in research, thereby being eager to learn it and take it in with no judments or prejudice.

Melissa: I've built collaboration through involvement with the Catalysis Innovation Consortium, which fosters collaboration between members of the pharmaceutical industry and academia.

Sivani: Meeting people and having discussions at conferences has really helped. But the challenge has been finding independent funding or time to work on these projects outside of funded project hours.

Susmita: Many of my collaborations have grown from early connections, either initiated during conferences or through shared research interests. During graduate school, I served as the president of a student society, which gave me the opportunity to invite and host numerous guest speakers. While my advisor supported the final list of invitees, I was the one actively reaching out, coordinating logistics, and building relationships. That experience gave me the confidence and network to approach researchers directly, and something that continues to serve me well as a PI. I've also built collaborations with national lab scientists when I identified strong technical overlap, especially in areas requiring specialized instrumentation or advanced modeling capabilities. I believe the PI plays a critical role in shaping a lab's collaborative culture. As a graduate student, I saw firsthand how important it is for advisors to encourage collaboration, not suppress it. That lesson has stayed with me – I now try to create an environment where students feel empowered to engage with external researchers and grow their scientific network.

1.3. What have you gained from these collaborations?

Hirogi: To be honest, many of my international collaborations have faced challenges, and I've learned important lessons from these experiences. For example, when drafting a paper, relying solely on email communication made it difficult to have productive discussions. I realized the importance of incorporating face-to-face meetings, even virtually. Additionally, I encountered issues with customs when shipping samples internationally – some valuable samples were held and never returned. Moving forward, I aim to minimize international sample transfers or find ways to ensure they meet customs requirements to avoid such losses. These experiences have helped me reflect on how to build more effective and resilient collaborations in the future.

Monika: I gained patience with - sometimes it takes time to work out the details so that everyone feels good with the process.

Azalea: Through these collaborations, I've gained new perspectives and expanded my technical knowledge. For example, I learned more about the photophysics of polymeric materials thanks to collaborators who were experts in that field. I've also developed project management skills by juggling multiple projects simultaneously which has proven essential throughout my PhD.

Krystyna: Each collaboration gave me something different: from co-authored publications to technical expertise that I would not have access to in my home lab. But beyond the science, I gained perspective. Working in different countries has taught me how to adapt, communicate across cultures and approach

problems from different angles. I also found a sense of belonging to an international scientific community. Being part of diverse teams made me feel that I was not just doing science in the world, but with it. From contacting labs to raising funding, I had to learn how to navigate systems, advocate for my ideas, and take responsibility for my path. That experience gave me more than research skills – it gave me confidence as a globally minded scientist. And honestly, that mindset shift might be the most valuable thing of all.

Katharina: I have gained valuable insights in the research fields of others, and even more important in the thought processes of other disciplines, which in turn has shaped my approach to problem solving..

Cel: I have gained many helpful insights on ways to improve research. Furthermore, these collaborations almost always form important relationships with colleagues who can serve as a sounding board later on. Diverse perspectives and collaboration has been a great way to shake things up with the traditional research process.

Diego: I have gain friends, mentors, and expanding my publishing scope.

Jianyu: New ideas, understanding of new fields, high-level publications.

Bryan: From my collaborations, we have not only been able to further our science , but we have also gained professional friendships.

Roxana: Fruitful collaborations have resulted in research being disseminated through peer reviewed research articles and platform presentations at international conferences.

Eleonora: First and foremost I have gained new friends beyond science who enabled be to be exposed and better understand different cultures. Then I develop a new way to look at the problems.

Melissa: This collaboration has allowed me to introduce my new research group to the synthetic and computational organic chemistry community. It has also provided a means for members of my research group to become exposed to a wider range of chemistry and to learn how to communicate their research in computational chemistry to experimental groups.

Sivani: Lots of really good insights into how other industries, disciplines, and even regulators work.

Susmita: One of the most valuable lessons I’ve learned through collaboration is the importance of humility, especially when venturing into new research areas. Early in my career, I often felt pressure to have all the answers, but over time I’ve realized that meaningful progress comes when you're unafraid to say, “I don’t know, but I’d like to learn.” In fact, when entering a new field, I’ve often felt like I knew less than an undergraduate, and that’s exactly when collaboration becomes most powerful. It allows you to lean on others' strengths, gain clarity faster, and avoid reinventing the wheel. From a technical standpoint, collaborations have given me access to expertise, instrumentation, and methodologies that my lab alone couldn’t provide. But beyond the science, they’ve helped me grow as a researcher, teaching me how to ask better questions, communicate more clearly across disciplines, and stay open to critical feedback. I tell my students: don’t let ego get in the way of learning. The willingness to reach out, ask for help, and genuinely listen is what turns a collaboration into a transformative experience.

2. Connecting science and society

2.1. How do you approach communicating science to colleagues from different disciplines? Have your collaborations informed this?

Hirogi: When communicating science to colleagues from different specialties or disciplines, I try to focus on the core concepts and the broader significance of the research, rather than technical details. I make an effort to use accessible language and analogies that resonate across fields.

Monika: It's important to understand the background of the person I am speaking with so that I can try to explain things in a way that will be easily understandable. For instance, the person I am speaking with may already be familiar with a method, or there might be a way where I can relate the topic to an everyday topic.

Croix: Effective communication in science to colleagues from different specialties needs to be high-level enough for scientific generalists to understand with clear objectives and success criteria. To me, PhD training was less about learning about a specific topic and more about learning the process and language of scientific inquiry. Most PhDs will understand a problem, the approaches to a problem, and what success looks like as long as the communicator adequately and effectively conveys the information at a high-level. This should be true regardless of whether the receiver possesses the domain-specific knowledge and context to understand the nuances.

Azalea: My approach has always been to minimize jargon, even when speaking with people in my own field. Collaborating with colleagues from different departments and specialties has reinforced the importance of clarity. Clear communication avoids misunderstandings and makes scientific discussions more productive.

Krystyna: Collaborating with people outside my subfield has made me more aware of how much jargon I use, and how often we assume that certain scientific concepts are common knowledge, when in fact they are not. I have learned to slow down, explain the “why” before the “how,” and focus more on the bigger picture. This is something I am still working on, especially when talking to people from different fields.

Katharina: I take care that we all speak the same "language", i.e. make sure I explain special terms from my field and in return ask many questions to my opposite. I found it best to directly see how the experiment works in person rather than talking about it for hours, as it is often only then that I truly understand where impasses occur ect.

Cel: My research is interdisciplinary in nature due to being at the nexus of chemical and biomedical engineering. This results in many interactions with people from all across STEMM, from biologists and medical doctors to chemists, materials scientists, and engineers with a variety of backgrounds. I recently presented a talk on the same project to an audience of tissue engineers and an audience of polymer chemists. I highlighted different aspects of the project to connect to the presumed existing knowledge base of each audience, while simultaneously aiming to "teach" them about the area that would be more novel to them. Years of working in this research area has made me better equipped to explain the science in an understandable way, but, I do think that this is a skill that I am still working to refine. There is a lot of hyper

specific jargon we can become bogged down in, and we sometimes may need to take a moment to recalibrate.

Diego: I focus on analogies, explaining principles with day-to-day activities. Yes, my collaborations has force me to translate my research to other fields, sometimes even using different terms.

Jianyu: Take the initiative to contact people from different fields/disciplines and deepen scientific exchanges through collaboration on a certain project. Yes, most of communicating science is via scientific collaboration.

Bryan: I tend to find a middle ground between how I would tell my parents what I do and how I would describe it in a research paper. It is not always successful on the first go around, but that is OK. Usually it is from the second or third time explaining that inspiration hits and novel ideas are had.

Roxana: Communicating science with colleagues from different disciplines can be challenging. I try to approach it by explaining the abstract or complex concepts of my research using digestible language that can readily be interpreted and avoiding technical jargon. Collaborations with scientists from different fields have informed my communication approach by providing different perspectives that can be leveraged. Learning from others' research and experience is insightful for my communication skills.

Eleonora: I try my best to wear someone'else shoes and understand the basic knowledge of those scientists on the research topic I am working on. I have also found that making analogies to common, real life episode so that people can better and easier relate to.

Melissa: My approach is to focus on communicating the big picture challenges that my research group is tackling. I communicate results in a manner that provides sufficient background information to enable a clear understanding of the data that I am presenting. Lastly, I focus on generating a story rather than including every last bit of technical detail. Theme and story are key to communication.

Sivani: One of the biggest challenges I've faced working in an interdisciplinary context is jargon. As scientists, we all have different jargon, but sometimes the same word can mean vastly different things in different disciplines. For example, the term "endpoint" in toxicology, life cycle assessment, or physical chemistry has different meanings. So one thing I try to do is no matter who my audience is define some of the key terms and how I am using them in my own work.

Susmita: Yes, my collaborations have strongly influenced how I communicate science across disciplines. I work in the area of batteries, which naturally overlaps with chemistry and materials science, so when I talk to colleagues in those fields, we can easily dive into details like interfacial reactions, material selection, or electrochemical mechanisms. But within my own department, which is more mechanically focused, I've had to adapt my communication style. There, the questions are often different, more focused on battery performance, thermal safety, mechanical failure, or lifetime modelling, rather than what materials go inside the cell or how interfaces behave at the molecular level. This shift taught me to reframe my work based on what's most relevant to the audience. Instead of focusing on the atomic-scale processes, I emphasize how our materials improve cycle life, reduce degradation under mechanical stress, or help build safer, more robust systems. These collaborations and internal conversations have trained me to think beyond my core expertise and present research through multiple lenses.

2.2. Have you had the need to make your science digestible for non-scientists? How did you do it?

Hirogi: Yes, I have had several opportunities to make science accessible to non-scientists. One of the most meaningful has been through my outreach activities on YouTube, where I introduce not only my own research but also the work of others. In these videos, I focus on the real-world relevance of the research and avoid technical jargon as much as possible. To convey complex ideas intuitively, I use simple analogies and visual aids. For example, in one short video, I explained the toughening mechanism of elastomers using sacrificial bonds with just paper and a pen, which helped viewers, even those without a background in chemistry, grasp the concept easily. I received feedback that it was clear and engaging. At the same time, I don't completely avoid scientific terms; instead, I briefly explain them in simple language to help non-scientists become more familiar with scientific vocabulary. These experiences have taught me that effective science communication is not just about simplification, but also about building understanding and curiosity.

Monika: Yes, definitely. I used to speak to middle school students about science and I currently am a STEM pen pal to middle school students. When I was in grad school I practiced science communication a lot, attended workshops, and participated in my university's "Three Minute Thesis" competition to explain my dissertation work to a lay audience within three minutes. It was a challenge, but I put a lot of work into in and ended up placing first out of 50 competitors!

Croix: Connecting your science to something observable and relatable to everyday life seems to work well. Of course, telling a good story is always paramount.

Azalea: Yes, I've presented my research to middle and high school students. To make the science more relatable, I focus on the big picture. For example, I describe my work on degradable semiconducting polymers from carotenoids by saying: "I use the natural pigment that makes carrots orange to develop components for electronic devices. These materials break down after disposal, reducing environmental impact." Making analogies to everyday life helps audiences connect with the science.

Krystyna: Definitely! Especially with my friends and family. I have learned that metaphors and apt comparisons can accomplish a lot. For example, when I talk about synthetic transporters of anions, I like to compare them to tiny vehicles that help charged species pass through oil-like membranes in our cells. I also try to share fragments of my research on social media, which really forces me to simplify without oversimplifying.

Katharina: Yes, we do a 'participation lab' for Kids and teenagers from elementary school to high school. These courses have taught me to adapt my language to the depth of knowledge I encounter.

Cel: I have! I have some experience teaching undergraduates and pre-college, as well as engaging in activities like Skype a Scientist and Letters to a Pre-Scientist. I remember once having to deliver a talk about my doctoral thesis work to an audience of kindergarteners, which was interestingly the most difficult talk I gave during my degree! The key is empathy – imaging what they know based on past experiences, and trying to connect to their existing knowledge base. Plus lots of fun hands on examples where applicable!

Diego: Yes, I participate in outreach activities. We organize game stations where the phenomenon is explained by other activities. For example, paper recycling can explain adhesion, or food coloring explain diffusion.

Jianyu: Occasionally, I need to introduce my research to parents, middle school students, high school students, and undergraduates. Explain my new discoveries and research through simple analogies or by using common phenomena and laws in daily life.

Bryan: All the time! In this case, I rely on analogies and similes. A lot of the time it comes down to vocabulary. It is not always easy, but figuring out a good one can go a long way. For example, my research focuses on plastics, which are the combination of polymer and additives. The similes I use is to compare plastics to pastas. Like pastas, polymers come in lots of different shapes and sizes. Like additives, pastas have lots of different sauces. Sometimes the similes can capture multiple concepts. In the pasta example, a clump of spaghetti is useful analogy to the entanglements of polymer chains.

Roxana: Yes, I have had to make my science digestible for non-scientists. For example, I participate in a mentorship program where I communicate my research with middle school students. This opportunity to explain my research in a simple manner has been challenging but inspiring. For this particular audience, I try to use simple terms and analogies to explain complex ideas. I also use illustrations to describe scientific concepts because it supports different learning styles.

Eleonora: I have had, especially to my mum and friends. As I have been working on drug discovery I found one of the easiest way to relate to my science was to use Lego analogies (building blocks) as way to better understand how and why I was combining blocks together to create new molecules and how these would engage and "click" with proteins/biological targets as a do keys with lockers.

Melissa: Yes, I have presented my research to a non-scientific community. I did this by teaching the audience key terminology and concepts using slides with visuals and minimal text. I also gave specific examples on the application of my research area to solving challenges that we face on a daily basis.

Sivani: When presenting to non-scientsts or even non-chemists, my first goal is to identify why they would be interested in my science and what I want them to take away from the science. Based on that, I can craft my presentation around making sure the underlying message is communicated without getting too bogged down with details. However, I find one of the biggest challenges with this is communicating uncertainty without discrediting the science.

Susmita: Yes, absolutely, and I believe it's a responsibility, not just a skill. Whether it's for broader impact statements, funding justifications, public engagement, or even talking to family members, I've had to explain my battery research to people with no technical background. What helps most is always asking: "Why does this matter to someone outside the lab?" I try to distil the core message by focusing on the real-world impact: safer electric vehicles, longer-lasting portable devices, or more resilient energy storage for the grid. Rather than getting into electrochemical terms or interfacial dynamics, I explain the challenge in terms of what people care about: faster charging, batteries that don't catch fire, devices that last longer. I also ask myself: "How would I explain this in two sentences to a high school student or a policymaker?" That forces clarity. Using relatable analogies, visuals, or comparisons, like thinking of a battery as a sponge or a traffic system, can make abstract science tangible.

2.3. What is the value of making science accessible to society?

Hirogi: Making science accessible to society is essential for building a more informed, empowered, and resilient public. When scientific knowledge is communicated clearly and openly, it enables people to make evidence-based decisions in their daily lives, from health and safety to environmental responsibility and technology use. It also fosters trust in science, encourages critical thinking, and helps bridge the gap between researchers and the communities they serve. Moreover, accessible science can inspire the next generation of scientists and innovators by showing that science is not just for experts, but something that belongs to and benefits everyone.

Monika: The value of making science accessible is that society then understands more about the value of science – how it relates to everyday items, medicines, industrial processes – but also that it's important to keep funding it, along with other educational opportunities for youth to participate in science programs, especially those from underserved populations.

Croix: Making science accessible to society simply provides more opportunity for those typically excluded to participate in science. There are many ways to make science accessible, writing and reading science fiction being one of them. Science fiction also stirs the imagination on what’s possible. I read once in a Scientific American article why scientists should care about science fiction: Kristen Koopman says, “It reaches more people than will ever read any scientist’s papers. And that, if for no other reason, is why scientists should care.” (https://www.americanscientist.org/blog/macroscope/to-boldly-know-why-scientists-should-care-about-science-fiction)

Azalea: Making science accessible has many benefits. First, it helps combat misinformation—people need access to accurate scientific information to make informed decisions. Second, accessible science fosters collaboration. Many of my own collaborations were made possible through the open dissemination of my research via publications and conferences.

Krystyna: Making science accessible builds trust. If people understand not just what we do, but why we do it and for whom, science becomes less distant and more relevant. Collaboration plays a big role here. When we work across disciplines, countries, and sectors, we learn how to listen better, adapt our message, and connect with broader audiences. Making science accessible isn't just a communication challenge – it’s a moral responsibility. If society funds our research and feels its impact, then it deserves to understand it. I believe that accessible science leads to informed decision-making, stronger public trust, and more inclusive innovation.

Katharina: Only if society understands what we are doing at universities, they will trust, accept and adapt our findings. Additionally, we owe it to the general public to share the outcome of projects which have often been funded publicly.

Cel: The more society understands science and its importance, the less separation they will feel from both the research itself and the scientists who conduct that work. Especially now, public perception of science can have an impact on law, funding, and other factors that then snowball and can impact the quality of forthcoming work. There needs to be a mutual exchange between science and society.

Diego: It helps to make people more critical, and helps seeing science as an activity rather than an clear and set answer

Jianyu: It is not only reflected in technological progress and economic development, but also permeates social culture, civic literacy and the sustainable development of human future.

Bryan: It is extremely valuable in terms of understanding the process of scientific discovery and the utility of those discoveries.

Roxana: Making science accessible to society fosters a well-informed world built on trust and equity. Accessible science is powerful: apart from advancing research progress, it can drive innovation, encourage curiosity, and create a better future.

Eleonora: Science, to be truly impactful, needs to be readily accessible and understandable by anyone, particularly those with no scientific background. I have found enjoyment in outreach activities and promoting science activities to young children in school. I have found these activities promoted creativity and inclusivity, empowering kids to understand life from a different and more "informed" point of view. Science is not a subject of "a few elite old white men with lab coats", but science can be done by anyone.

Melissa: Making science accessible to society is critical because it provides the public with insight on the importance of investing in science and it expands the diversity of individuals who become scientists.

Sivani: I believe that understanding the scientific method improves critical thinking and trust in science. Making science accessible to society also helps to underscore the value of science. When things are in a "black box" or up in an "ivory tower", it is hard for people to see how science is affecting their day-to-day lives and how investing in public and fundamental science is important.

Susmita: Making science accessible is fundamental to societal growth and progress. I agree with the common critique that many scientists are poor communicators, and I believe this needs to change. Communication skills should be an essential part of graduate curricula so that scientists are better prepared to explain their work beyond academic circles. When society understands scientific knowledge, it creates a more informed public that can support important initiatives, whether that’s investing in clean energy technologies or addressing climate change. If people grasp the evidence behind global warming, for example, we wouldn’t still be debating its reality; instead, we could focus on solutions. Accessible science builds trust, drives policy, and ultimately empowers communities to participate meaningfully in decisions that affect their future. For science to truly benefit society, communication can’t be an afterthought – it must be integrated into the way we train scientists and share discoveries.

3. The future of collaboration – a call for action

3.1. What are you doing to create collaborations?

Hirogi: To create collaborations with early-career scientists, I actively participate in academic conferences and exhibitions. These events provide valuable opportunities to meet researchers from diverse backgrounds, exchange ideas, and explore potential joint projects. I find that informal conversations during poster sessions or networking events often lead to meaningful connections and future collaborations.

Monika: I am part of a wide network of science and technology policy fellows who pass along opportunities to each other and serve as mentors to each other. I also serve as a STEM pen pal to middle school students to open the thinking about pursuing STEM related studies in the future.

Azalea: Through networking at conferences and online, I actively pursue collaborative opportunities—both research-related and beyond. For instance, I'm involved with the Canadian Society for Chemistry's Working for Inclusion, Diversity, and Equity (WIDE) committee. I regularly collaborate with others to advance equity, diversity, and inclusion (EDI) within the Canadian chemistry community. For example, I've worked with Jacky Deng (CAS Future Leader, Class of 2022) on several EDI initiatives through WIDE, including the Scientific Spotlight video series highlighting underrepresented groups in chemistry, and a quarterly webinar series focused on EDI-related topics.

Krystyna: This editorial is a great example in itself – a peer-led project that brings together future leaders across continents, without institutional support, hierarchy, or formal funding. It's been a unique way to collaborate as equals, driven by shared purpose rather than obligation. Beyond that, I stay in touch with colleagues from my research internships. While many of us are still early in our careers, often without the resources or authority to launch independent collaborations, we're laying the groundwork. We exchange ideas, support each other's work, and keep our networks warm so that when opportunities do arise, we're ready to build together. Early-career collaboration starts less with formal projects and more with curiosity, trust, and informal conversations.

Katharina: The secret ingredients in my opinion are to find true win-win situations for all involved, talk often, and be patience.

Cel: I create collaborations mainly by aiming to discuss with others working in a similar area, or even a distant area. This can happen in a variety of ways, but often is due to us being in some environment and having some unstructured time to network, so at a conference, meeting, or other gathering. Occasionally, events that are purely social in nature but for common groups of academics (e.g. events I have hosted with my Stanford LGBTQ+ Postdocs group) have also produced some fun collaborations with fellow early career researchers! I have also had some productive discussions with fellow Future Leaders to seek advice, such as asking for feedback on faculty applications, funding, and postdoctoral research.

Diego: I organize symposia with them, or invite them to be committee members in my students so they can get involved

Jianyu: Seek collaboration in person at academic conferences and in daily research, especially with young scholars and future leaders in emerging technologies (e.g., AI, ultra-fast spectroscopy)

Bryan: I stay on top of my email. You would be surprised how many cold emails you receive that go unanswered. I answer them! It goes a long way and usually if someone takes the time to write, they are motivated to do something. I also keep up with colleagues I have met at workshops and events. It is not every week, but every quarter or semester a quick check-in can do wonders. Practically, when I am thinking about the next proposal I am going to write, I spend some time considering who in my network has expertise that would make the proposal that much more exciting, and then I get them on Zoom to see what we could do together.

Roxana: To create collaborations with early career scientists/Future Leaders, I identify potential collaborators to networking with at conferences I plan to attend. During the conferences I discuss ideas or potential projects that we could work on together. I then follow up post-conference through email or Zoom chat to continue our discussions and plans.

Eleonora: Although I am working in a corporate environment and "collaborations" outside this environment sometimes are a bit overlooked due to business/confidentiality matters, I have still kept contact with most of my former colleagues and friends to keep involved in their research and new project so to seek also possible business opportunities/new collaborations.

Melissa: I am a member of the Catalysis Innovation Consortium, which brings together individuals from the pharmaceutical industry and academia. There are some early career investigators who are part of this organization. I am also a member of the University of Minnesota Chemical Theory Center, which provides me with the opportunity to collaborate with early career PI's in theoretical and computational chemistry.

Susmita: In my classes, I design group projects that mix students at different levels to encourage peer learning and engagement across experience levels. I actively encourage students to discuss their research openly with others rather than keeping it to themselves, fostering a culture of sharing and collaboration early on. Outside the classroom, I maintain contact with other early-career researchers I've met through conferences and professional meetings. I regularly explore potential opportunities for collaboration, whether through joint grant proposals, co-mentorship, or shared experiments. As I recently completed my first year as an independent professor, I am planning to expand these efforts further, building a network of future leaders who can support and challenge each other. I see this as a critical part of my growth as a PI and my commitment to fostering a vibrant and collaborative research community.

3.2. What could you and society win from these collaborations?

Hirogi: Collaborations with early-career scientists offer mutual benefits. For me, they bring fresh perspectives, new ideas, and the opportunity to grow through interdisciplinary exchange. These interactions often challenge my assumptions and inspire innovative approaches to research. For society, such collaborations help accelerate scientific progress by combining diverse expertise and fostering the next generation of researchers. They also promote a more inclusive and dynamic scientific community, which is essential for addressing complex global challenges.

Azalea: These collaborations promote chemistry as a viable and rewarding career for underrepresented groups, helping to build a more inclusive scientific community. Increased diversity brings new ideas and approaches, which fosters innovation and benefits society as a whole.

Katharina: More outcome with the same amount of resources.

Cel: One of the biggest lessons I have had to learn is that science is not a solitary endeavor – you can't go it alone. Especially for people who are underrepresented in STEM, sometimes it can feel that this is our only option (for example, some folks will not want to collaborate with certain demographics due to preconceived notions). It is especially important to cultivate a network of mentors, but also to establish some form of community of peers. This can be a challenge and is no small feat, but this network is a great structure that will make the research more streamlined and effective. For example, there's no use teaching yourself a new skill for months when your colleague who is a leading expert can have the results to you by the end of the week. It is evident how much more and better science we can do this way, which ultimately benefits society as well.

Diego: I think that you short the generational gap, helping to communicate with new language that can then be easier to translate to the society.

Jianyu: Together we can solve a scientific problem and promote scientific progress; we can further spread scientific knowledge in different fields; we can generate new scientific viewpoints and perspectives in cooperation.

Bryan: Better science and a better understanding of each other.

Roxana: As an early career scientist/Future Leader myself, I gain fresh perspectives, networks, and community from collaborating with other early career scientists/Future Leaders. Society benefits from collaborations driven by early career scientists/Future Leaders through groundbreaking innovative science and non-leaky STEM pipelines that prepare the next generation of scientists and leaders that make our world safer and more sustainable.

Eleonora: I believe that bridging the gap between industry and academia, or better combining both strengths, would be a great asset to society. Academia would benefit from a more hands-on practical approach to research characterising industry, focused on deliverables with a possible bigger impact, owing to business interests (e.g., commercialisation of research) and bit broader means availabilities (cutting-edge technologies equipments). On the other side, the multicultural, more diverse background, age of teams and researchers working in academia could benefit industry to address scientific challenges with a more fresh approach, like from those who have not many years of research experience behind or are completely new to.

Melissa: Together, we could more efficiently generate new solutions to the biggest challenges in society, such as those related to climate change, sustainability and human health.

Susmita: In many fields, research often progresses incrementally, building carefully on existing knowledge. That makes it especially important to engage with a diverse group of collaborators and actively seek fresh perspectives. If we only talk to the same people within our immediate circles, we risk limiting innovation and overlooking new ideas. Collaborations with early-career scientists bring in different viewpoints, challenge assumptions, and help break out of echo chambers. This diversity fuels creativity and can lead to breakthroughs that wouldn’t emerge otherwise. For society, these collaborations accelerate progress on critical challenges such as developing safer, more efficient batteries and trying to address water

issues, which are essential for sustainable development and public health. By nurturing a broad, inclusive network of future leaders, we also strengthen the scientific workforce capable of tackling these urgent issues and creating lasting impact.

3.3. Dreaming big: Building your network (What ideas do you have to build new collaborations? What support would make that possible?)

Hirogi: To build new collaborations, I envision creating platforms that connect researchers across disciplines and career stages, such as interdisciplinary workshops, online forums, or collaborative video projects. I'm particularly interested in using science communication tools, like YouTube or webinars, not only to share research but also to spark dialogue and co-creation among scientists and non-scientists alike. Support in the form of funding for travel, digital infrastructure, and mentorship programs would be invaluable. These resources would help facilitate meaningful exchanges, especially with international partners, and foster a more inclusive and innovative research network.

Monika: I am just starting a new role and am certainly in network-building mode now. I am not sure what support I would need at this point. I am already deep in new collaboration stage :)

Croix: Attend more conferences and write publicly (e.g., blogs, social media). I personally need to do more of both, but these seem like good starting points.

Azalea: More small-scale, student-centered professional development workshops and symposiums would be immensely beneficial. Often, PhD students are expected to navigate large conferences with little preparation, which can be overwhelming. Organizing more accessible, department-level events across multiple scientific specialties (ie. organic chemistry, theoretical chemistry, analytical chemistry) would help students develop the skills and confidence to network effectively.

Krystyna: Research stays, internships, and mobility programs are invaluable. They give you a chance to get to know someone as a person – and that human connection is what builds real trust. You can read someone's papers for years, but sharing a lab bench or a coffee break changes everything. I would love to see more programs that not only initiate collaborations but also help sustain them through follow-up meetings, long-term project support, or simply space for continued scientific dialogue. Collaboration must not end with a single paper and a polite goodbye. The best ideas grow slowly; they need time, continuity, and people willing to keep the conversation going.

Cel: Probably, there will be more opportunities coming for international collaborations and ways to use technology to streamline collaborative work. For example, it would be nice to have some sort of interactive database to look up individuals based on specific skillset or potential contributions.

Diego: I think giving support for social activities in conferences, or just invited seminar cycles would help people to talk more and build ideas. Giving funds for starting these ideas could really help cement collaborations.

Jianyu: Opportunities to communicate with researchers from different disciplines (such as through X, international academic conferences, or even email communication). I think I need to have something unique (such as skills or equipment that the other party does not have), new ideas, etc.

Bryan: We need to rethink the scientific conference to be more like a workshop where you have structured activities that get people interacting with each other rather than mingling over coffee. To make it possible requires dedicated professionals, not faculty volunteering their time. That was what made the CAS Future Leaders program so impactful.

Roxana: While my work with agricultural nanotechnology has so far involved collaborations exclusively with scientists, I recognize a critical gap between my research laboratory and real-world farms. My goal is to develop collaborations with farmers and farming communities to co-develop sustainable agro-nanotechnology. This would facilitate the practicality and translation of these technologies in agriculture.

Eleonora: I have found that a good way to build new collaborations is through social media, such as LinkedIn or IG (scientific content creators). Moreover, the old style "attending national or even better, international conferences is a great way to forge new collaborations".

Susmita: Looking ahead, I want to build a diverse, interdisciplinary network that bridges academia, national labs, and industry to tackle complex challenges in energy storage and environmental sustainability. One specific idea is to participate in and help organize conferences that are outside my dedicated field, interdisciplinary or multidisciplinary gatherings, or at least advocate for dedicated segments within existing conferences that encourage cross-field interactions. These venues provide fertile ground for meeting researchers with fresh perspectives and complementary expertise. I also see great opportunity in creating joint training programs or co-mentorship models with national labs and other universities, providing my students and postdocs with broader exposure and collaborative experience. To realize these ambitions, support such as seed funding for pilot projects, institutional encouragement for interdisciplinary partnerships, and administrative assistance for organizing events would be invaluable. Access to shared facilities and computational resources would further enhance the impact of collaborations.

Anything else you would like to add?

Hirogi: Above all, I'm excited to keep learning, collaborating, and sharing science in ways that connect with both experts and the broader public. Whether through research, outreach, or creative communication, I hope to contribute to a more curious, informed, and inspired society. I also want to express my sincere appreciation to those who created this questionnaire—it's thoughtfully designed and has given me a valuable opportunity to reflect on my experiences and aspirations.

Jianyu: I would like to share a sentence I saw recently and my thoughts: "Because we always believe that science has no borders, strengthening scientific exchanges is conducive to the growth of human knowledge and technological progress. Unfortunately, a few countries and institutions have tried to prevent normal international academic exchanges and study abroad cooperation in the name of national security. This wrong practice is neither timely nor popular, and is doomed to fail." It should be pointed out that the CAS Future Leaders project is a project that breaks this kind of normal academic exchange. I believe that my companions and I will benefit from it.

SURVEY PARTICIPANTS

Table 1. Detailed information on survey participants

Name	City/country	Area of chemical science and engineering	Current role or position	Career plans or goals
**Hirogi Yokochi**	Tokyo, Japan	Polymer chemistry	Industry scientist	Industry/Academia
**Monika A. Roy**	Boston, MA, USA	Environmental policy	Senior Director of Environmental Justice, Massachusetts Department of Conservation and Recreation	I plan to stay in the science policy space, working in government
**Croix Laconsay**	Seattle, USA	Computational chemistry and material science	Industry Scientist	Industry
**Azalea Uva**	Toronto, Ontario, Canada	Polymer chemistry	Industry scientist	Industry
**Krystyna Maslowska-Jarzyna**	Warsaw, Poland	Supramolecular chemistry, organic chemistry	Postdoc, Senior Assistant	Academia
**Katharina Ehrmann**	Vienna, Austria	Polymer chemistry, photochemistry	Assistant Professor	Academia
**Cel Welch**	Palo Alto, California, USA	Chemical engineering / biomedical engineering - flexible bioelectronics	Postdoc	Leaning towards academia, also considering industry
**Diego Gomez-Maldonado**	Texas, USA	Materials	Assistant Professor	Academia
**Jianyu Zhang**	Hangzhou, China	Materials science, photophysics	Tenure-track Assistant Professor	Academia
**Bryan James**	Boston, USA	Green polymers/chemical engineering	Assistant Professor	Academia
**Roxana Coreas**	Berkeley, CA, USA	Chemical engineering – nanotechnology	Postdoc	Academia
**Eleonora Comeo**	Arezzo, Italy	Drug discovery, herbal-based, natural products	Scientific Project Manager	Hold strategic positions (CSO) in pharma or healthcare R&D and deliver tangible solutions to patients
**Melissa Ramirez**	Minneapolis, MN	Organic synthesis and computational chemistry	Assistant Professor	Secure tenure and start my own company centred on drug discovery
**Susmita Sarkar**	Raleigh, North Carolina, USA	Electrochemical energy storage	Assistant Professor	Academia
**Sivani Baskaran**	Canada	Environmental chemistry	Postdoc	Industry/Academia

APPENDIX

Programs that support researcher mobility and international collaboration

(Based on the authors' experience)

- ERASMUS+, https://erasmus-plus.ec.europa.eu/.
- Polish National Agency for Academic Exchange (NAWA, long-term outgoing mobility for Polish researchers and incoming international researchers to Poland), https://nawa.gov.pl/.
- Foundation for Polish Science (FNP, prestigious stipend for early-career researchers, funding for research teams), https://www.fnp.org.pl/component/fnp_pages/.
- The Kosciuszko Foundation (Exchange Program to the US and to Poland), https://thekf.org/.
- Polish University-based programs (IDUB – Initiative of Excellence – Research University; ZIP / POB / Academic Excellence Centers – internal mobility and research grants, varies by institution).
- L'Oréal–UNESCO For Women in Science, https://www.forwomeninscience.com/.
- Fulbright Program (academic exchanges between Poland and the US), https://fulbright.edu.pl/, https://us.fulbrightonline.org/.
- COST Actions (funding for short-term scientific missions and network-building across Europe), https://www.cost.eu/cost-actions-event/browse-actions/.
- Marie Skłodowska-Curie Actions (MSCA, Horizon Europe), https://marie-sklodowska-curie-actions.ec.europa.eu/.
- Austrian University-internal funding (Dr. Maria Hörbiger award for young researcher mobility).
- Chemistry Europe Travel Grant (jointly awarded by the Austrian Chemical Society GÖCH and Chemistry Europe), https://www.chemistryviews.org/chemistry-europe-travel-grant/.
- International Younger Chemists Network (IYCN, enhancement grant), https://www.iycnglobal.com/.
- Schmidt Science Fellowship, https://schmidtsciencefellows.org/.
- CONACYT (Mexico), https://www.gob.mx/conacyt.
- Leading Edge Fellows Program, https://www.leadingedgesymposium.org/.
- Rising Stars Programs (e.g. MIT Rising Stars in Chemical Engineering), https://cheme.mit.edu/rising-stars/.
- NCFDD Faculty Success Program, https://www.ncfdd.org/programs/faculty-success-program/.
- National Natural Science Foundation of China, https://www.nsfc.gov.cn/english/site_1/index.html.
- RGC Junior Research Fellow Scheme, https://www.ugc.edu.hk/eng/rgc/funding_opport/jrfs/.
